# Neuromuscular Control and Motor Performance Across the Menstrual Cycle in Physically Active Young Females

**DOI:** 10.1002/ejsc.70174

**Published:** 2026-04-11

**Authors:** Mareike Sproll, Nele Otterbach, Astrid Zech

**Affiliations:** ^1^ Department of Human Movement Science and Exercise Physiology Institute of Sport Science Friedrich Schiller University Jena Jena Germany

**Keywords:** motor control, performance, strength

## Abstract

This study aimed to analyze potential menstrual cycle‐related changes in physical parameters associated with performance and injury risk, focusing on neuromuscular control and motor performance in physically active young females. Twenty‐two healthy participants with regular menstrual cycles (24–34 days) were examined sequentially during the follicular phase (day 3), ovulatory phase (within 48 h after a positive urine LH test), and luteal phase (7 days post‐ovulation). Ovulation was indicated by a positive urinary LH surge test. The assessments included countermovement jump (CMJ), squat jump (SJ), postural control, ankle dorsiflexion range of motion (ROM), and isokinetic concentric maximal strength. Statistical analyses involved one‐way repeated‐measures ANOVA or the Friedman test for non‐normally distributed data. Significant effects across the menstrual cycle were found for maximum concentric flexion strength (*p* = 0.003; $\eta_{G}^{2}$ = 0.037) and ankle ROM (*p* = 0.043; $\eta_{G}^{2}$ = 0.010). Post hoc analysis revealed a significant increase in concentric flexion strength from the follicular to the luteal phase (*p* = 0.004), whereas no significant pairwise differences were observed for ankle ROM. Concentric flexion strength increased significantly from the follicular to the luteal phase (+7.4%), and ankle ROM showed a trend toward improvement, with the highest values observed in the luteal phase (+3.8%). In contrast, CMJ, SJ, and postural control remained constant across all phases. Overall, neuromuscular and motor performance parameters appear largely consistent throughout the menstrual cycle, with only small fluctuations in strength and flexibility. These findings suggest that menstrual cycle‐related changes have limited functional relevance but may still warrant consideration in future studies investigating individual responses and injury risk.

## Highlights

- Neuromuscular and motor performance parameters remained largely stable across the menstrual cycle, including jump performance and postural control.
- Assessments followed a fixed sequential order (follicular, ovulatory, luteal phases; no randomization) due to natural cycle progression.
- Small but statistically significant effects across the menstrual cycle were observed for concentric flexion strength and ankle dorsiflexion ROM. A significant post‐hoc difference was detected only for concentric flexion strength, with higher values in the luteal phase.
- The observed effects across the menstrual cycle were overall small, suggesting that their functional relevance for training and performance may be limited.

## 1 Introduction

Elite female sports performances have improved over the last century, but it is widely assumed that a considerable gap between female and male athletes will persist (Cheuvront et al. 2005). This difference is often attributed to sex‐specific biological factors, including muscle strength, aerobic capacity (Thibault et al. 2010), injury risk (Zech et al. 2022), and biomechanics (Kim et al. 2021). Among these, hormonal fluctuations in females play a key role, influencing physical performance as well as the structural and functional properties of tissues such as muscles, tendons, and ligaments (Hansen and Kjaer 2016).

Sex hormones affect human physiology, particularly testosterone, estrogen, and progesterone. Testosterone, with its well‐documented anabolic properties, drives the sex divergence in muscle mass and strength observed from puberty onward (Clark et al. 2019). Testosterone levels in men are four to five times higher than in females, explaining the divergence in performances that peaks during puberty and plateaus after that (Clark et al. 2019; Handelsman 2017). Estrogen and progesterone also influence strength performance and exercise response, though less significantly than testosterone (Wolf et al. 2012). Females experience unique hormonal fluctuations during the menstrual cycle, where estrogen and progesterone levels vary considerably. Estrogen peaks shortly before ovulation and remains elevated in the luteal phase (Hansen and Kjaer 2016). These changes affect tissue structures and adaptations, suggesting menstrual cycle‐related hormonal fluctuations impact performance, exercise adaptability, and injury risk in female athletes (Bell et al. 2014; Hansen and Kjaer 2016).

The impact of hormonal fluctuations on athletic performance and injury risk has been extensively debated. Systematic reviews and meta‐analyses, such as McNulty et al. (2020), suggest that the effects of menstrual cycle phases on exercise performance are generally minimal. Similarly, Meignié et al. (2021) found no evidence for changes in jumping and sprinting performance across the menstrual cycle. However, specific studies indicate hormonal fluctuations may influence ligament laxity, postural control, and muscle strength, potentially affecting injury risk (Hewett et al. 2007; Thompson et al. 2021). For instance, ACL injuries are reported more frequently during the preovulatory phase, possibly due to increased estrogen levels altering neuromuscular control and joint stability (Hansen and Kjaer 2016; Herzberg et al. 2017; Hewett et al. 2007).

Despite growing research, significant gaps remain. Many studies focus on isolated performance or injury risk aspects, such as endurance or ligament laxity, without considering interactions between multiple neuromuscular and biomechanical factors. Evidence on strength adaptations is inconsistent, with some studies reporting greater strength in the follicular phase (Thompson et al. 2021) and others finding no significant differences across the cycle (McNulty et al. 2020; Meignié et al. 2021). Although it seems likely that the observed changes are a result of a complex interaction of several risk factors, including increased ligamentous laxity, unfavorable lower‐limb alignment, impaired neuromuscular control, and reduced muscular strength, previous research has typically assessed these factors separately rather than in an integrative manner, even though all of them have been implicated as contributors to ACL injury risk in female athletes (Hickey Lucas et al. 2017; Myer et al. 2006; Smith et al. 2012). Therefore, the objective of this study was to take a comprehensive look at multiple neuromuscular control parameters at crucial stages of the menstrual cycle to analyze whether and to what extent their potential changes should be more closely monitored for the prevention of major lower extremity injuries in females.

## 2 Materials & Methods

### 2.1 Study Design

An observational study with a within‐subject design was employed.

### 2.2 Participants

The study participants were recruited by distributing flyers in the university environment and local sports clubs. Females between the ages of 18 and 35 with a self‐reported regular menstrual cycle were included in the study. Female participants who could not rule out pregnancy, who had taken hormonal contraceptives in the last 6 months, who had current lower limb injuries, who had an irregular menstrual cycle, or a cycle length of less than 21 days or more than 35 days were excluded from the study. All participants were fully informed about the study and had given their written consent. Each female participant voluntarily agreed to take part in the study and was explicitly informed that they could withdraw at any time without giving a reason. The study was approved by the local ethics committee (FSV 22/010) and conducted following the Declaration of Helsinki.

### 2.3 Menstrual Cycle Monitoring

Three measurement times (Sessions) were set at the follicular (Session 1), ovulation (Session 2), and luteal phases (Session 3). The follicular phase was defined as cycle day 3 (2 days after the onset of bleeding). Ovulation was determined using a commercially available urinary luteinizing hormone (LH) test (Loop, ESTRADE, Germany). As per the manufacturer's instructions, participants initiated LH testing individually between the 5^th^ and 12^th^ day of the menstrual cycle. The test was performed at about the same time of day until the afternoon at the latest. Test results were documented photographically and verified by the investigators. The ovulatory phase was defined as occurring within 48 h after the positive LH test result, and the luteal phase as 7 days after ovulation. The 21–35 days range describes a regular cycle, so we asked the females about their prior cycles to include only females with this cycle length in the study (Goldsmith and Glaister 2020; Thompson et al. 2021). If the urine LH test remained negative until cycle day 20, the ovulation and luteal phase measurements were postponed to the participant's next menstrual cycle to ensure accurate phase classification (Draper et al. 2018).

### 2.4 Procedure

All testing sessions were scheduled at comparable times of day to minimize circadian variability and followed an identical protocol. Anthropometric and body composition measures were obtained using bioelectrical impedance analysis (InBody 720, InBody, Eschborn, Germany). Female participants then completed a standardized warm‐up on a cycle ergometer before undergoing the test battery, which comprised standing postural sway, jump performance, agility, and concentric maximum strength assessments. Leg dominance was determined by asking participants which leg they preferred for kicking a ball. To reduce potential confounding by acute fatigue or hormonal variation, female participants were instructed to abstain from high‐intensity exercise for 24 h before each session. Compliance was assessed via self‐report using a questionnaire in which female participants confirmed adherence to this requirement.

### 2.5 Vertical Jump Tests

The explosive power was tested with the countermovement jump (CMJ) and squat jump (SJ). The CMJ was performed from an upright stance (feet hip‐width apart) down to a self‐selected squat position and then to the maximum jump height. During the SJ, participants squatted to a depth of 90° knee flexion and held this position for three seconds. A goniometer (Sport Tec, Pirmasens) was used to ensure the depth before they jumped as high as possible. Both jumps were performed with the usual sports shoes and hands on the hips. Each subject completed three repetitions of each jump condition after three familiarization trials, with the highest jump being used for further analysis. The jumps were performed on the 3D force plate (Kistler type 9260AA6, Kistler, Germany) and the jump height was determined using the MARS software (Kistler, Germany).

### 2.6 Static Balance (Body Sway)

Balance was measured using body sway on a force plate (Kistler, Germany) in a semi‐tandem stance, with the toes of the non‐dominant foot positioned next to the inside of the dominant heel. The body's center of gravity was shifted to the contralateral leg, with the dominant leg continuing to be weighted and remaining extended. The hands were placed on the hips. Female participants focused on a fixed point on the wall to align their head position and line of vision before closing their eyes for the trial. During each trial, participants had to stand as motionless as possible for 20 s. After three familiarization trials, participants completed three evaluation trials. The sway path was recorded with MARS software (Kistler, Germany), and the best trial (smallest path) was used for further analysis.

### 2.7 Ankle Dorsiflexion Range of Motion (ROM)

The range of motion of ankle dorsiflexion was determined using a modified weight‐bearing lunge test (WBLT; Cejudo et al. (2014)). Female participants stood barefoot in a lunge position with the non‐dominant leg on a wooden box (29 × 55 × 40 cm). The dominant leg, which was used for the measurement, stood on a measuring tape positioned on the floor directly below the body's center of gravity. A metal bar, positioned orthogonally to the box, was used to ensure the alignment of the contralateral leg at the level of the lateral malleolus and the head of the fibula. An inclinometer (Fabrication Enterprises Inc., New York) was placed with the upper end attached to the tibial tuberosity to measure the achieved dorsiflexion range. Participants were instructed to maximally push the knee of the contralateral leg forward without lifting the heel. The maximum distance achieved was recorded. Each female participant completed three repetitions after three familiarization trials, and the best trial was used for further analyses.

### 2.8 Isokinetic Maximum Strength

The maximum concentric strength of the knee extensor and flexor muscles was measured using an isokinetic dynamometer (IsoMed 2000, D. & R. Ferstl GmbH, Germany) in a seated position. The seat, adapter length, and device pivot point (knee joint gap) were adjusted to each participant's leg dimensions, ensuring the knee and hip angles were approximately 90°. The adapter was attached just above the ankle joints, and the non‐dominant thigh, pelvis, and shoulders were secured to prevent movement. The range of motion ranged from 15° to 90° of knee flexion. After ensuring the full range of motion, participants completed three familiarization sets at an angular velocity of 60°s^−1^ to ensure a smooth transition between flexion and extension followed by a 60‐s rest. Then they completed three maximum concentric contractions at the same angular velocity, with the maximum torque used for further analysis.

### 2.9 Statistics

The statistical analysis of the results was carried out using the software RStudio Version 4.0.3 (RStudio, Boston). The data were tested for normal distribution using the Shapiro‐Wilk test and for homogeneity of variance using the Levene test. If these requirements were met, a one‐way ANOVA with repeated measures and subsequent post‐hoc comparisons with Bonferroni‐Holm correction were performed. The condition of sphericity was also calculated with the Mauchly test in the ANOVA, and in case of a violation, the Greenhouse‐Geisser correction was used. The results were subsequently presented with the mean and standard deviation (SD). Non‐normally distributed data were analyzed using a Friedman test, with results indicated as medians and ranges. If a significant overall difference was found, pairwise comparisons were performed using the Wilcoxon signed‐rank test with a Bonferroni correction to adjust for multiple comparisons. In addition to significance testing, effect sizes were calculated as generalized eta squared ($\eta_{G}^{2}$) for ANOVA (Bakeman 2005) and as Kendall's W (W) for the Friedman test (Tomczak and Tomczak 2014). The significance level was set at *p* < 0.05.

## 3 Results

Of the 33 female participants initially recruited, 11 were excluded from the analysis because they could not record ovulation or had irregular menstrual cycles. The 22 healthy females included in the final data analysis had a mean age (± SD) of 23.5 ± 3.9 years (range: 18–34 years) and a mean height (± SD) of 168.2 cm ± 5.0 cm. These females reported participating in sports activities for an average of 1.20 h (± 1.15 h) daily. Their menstrual cycles ranged from 24 to 34 days with a mean cycle length of 28.41 days ± 2.68 days. The majority (77%; *n* = 17) were right‐leg dominant, while the remaining participants (23%; *n* = 5) were left‐leg dominant. Of these 22 female participants, 2 (9%) failed to detect an LH surge in the planned cycle, requiring Sessions 2 and 3 to be rescheduled to the subsequent cycle while maintaining the fixed sequential order (follicular, ovulatory, luteal phases).

A complete summary of all results is provided in Supporting Information S1: Table 1 (Appendix).

### 3.1 Vertical Jump Tests

**FIGURE 1:**
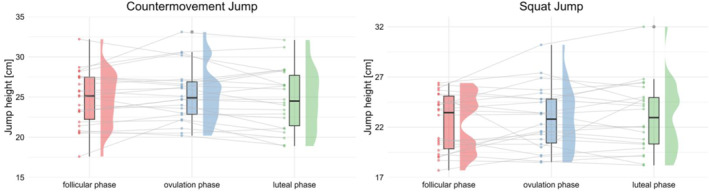
Jump performance data across the menstrual cycle. Violin plots show jump height [cm] for the countermovement jump (left) and squat jump (right) across the follicular phase, ovulation phase, and luteal phase. Box plots represent the median and interquartile range, with individual data points shown as dots. No statistically significant differences were observed between phases.

There were no significant differences between the phases of the menstrual cycle in the CMJ and SJ (Figure 1), with CMJ measurements of 24.8 cm ± 3.5 cm during the follicular phase, 25.4 cm ± 3.5 cm during the ovulation phase, and 24.7 cm ± 3.8 cm during the luteal phase (ANOVA: *p* = 0.224; F(2,42) = 1.55; $\eta_{G}^{2}$ = 0.007). Similarly, the SJ measurements were 23.45 cm (median, range: 17.7–26.4 cm) during the follicular phase, 22.80 cm (median, range: 18.5–30.2 cm) during the ovulation phase, and 22.95 cm (median, range: 18.2–32 cm) during the luteal phase (Friedman test: *p* = 0.719; χ^2^(2) = 0.659; *W* = 0.015).

### 3.2 Static Balance (Body Sway)

The body sway in the semi‐tandem stance was nearly identical across the phases: 473.05 mm (median, range: 298.30–901.00 mm) in the follicular phase, 478.65 mm (median, range: 310–777.30 mm) in the ovulatory phase, and 500.30 mm (median, range: 282.80–739.60 mm) in the luteal phase. The Friedman test showed no statistically significant effects (*p* = 0.580; χ^2^(2) = 1.091; *W* = 0.025).

### 3.3 Ankle Dorsiflexion ROM

**FIGURE 2:**
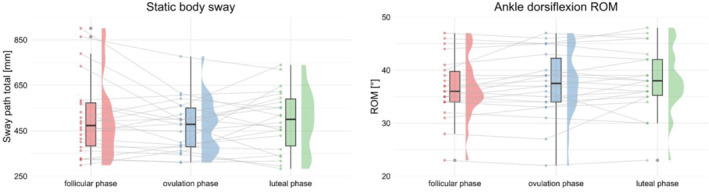
Postural control and ankle dorsiflexion ROM data across the menstrual cycle. Violin plots show sway path total [mm] for the postural control (left) and ROM [°] for the ankle dorsiflexion Range of Motion (ROM) (right) across the follicular phase, ovulation phase, and luteal phase. Box plots represent the median and interquartile range, with individual data points shown as dots. Postural sway did not differ significantly between phases. Ankle dorsiflexion ROM showed a significant main effect in ANOVA, but post‐hoc comparisons revealed no significant differences between individual phases.

The average values (±SD) for the ROM of the ankle show a gradual increase from the follicular phase to the luteal phase: follicular phase: 36.68° ± 5.78°, ovulatory phase: 37.50° ± 6.33°, luteal phase: 38.09° ± 6.05. The ANOVA showed a significant increase in ROM during the cycle (*p* = 0.043; F(2,42) = 3.40; $\eta_{G}^{2}$ = 0.010). The post‐hoc analysis with Bonferroni‐Holm correction showed no significant differences for pairwise comparisons, although there was a trend toward higher ROM in the luteal phase compared to the follicular phase (*p* = 0.072; +3.8%). Both results, static balance (body sway) and ankle dorsiflexion ROM, are presented in Figure 2.

### 3.4 Concentric Maximum Strength

**FIGURE 3:**
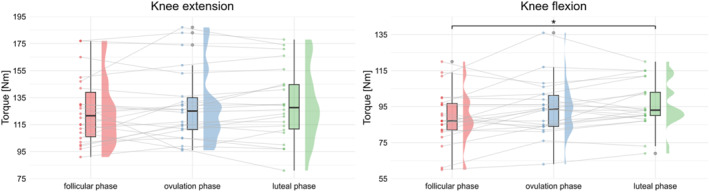
Maximum knee muscle strength data across the menstrual cycle. Violin plots show Torque [Nm] for the knee extension (left) and knee flexion (right) across the follicular phase, ovulation phase, and luteal phase. Box plots represent the median and interquartile range, with individual data points shown as dots. Knee extension did not differ significantly between phases. Knee flexion showed a significant difference in post‐hoc comparisons between the follicular and luteal phases (*p* = 0.004, indicated with * in the figure).

A significant effect of the menstrual cycle was found for concentric maximum flexion strength (ANOVA: *p* = 0.003; F(2,42) = 6.61; $\eta_{G}^{2}$ = 0.037). The post‐hoc test with Bonferroni‐Holm correction showed a significant difference (*p* = 0.004; +7.4%) between the luteal (95.91 Nm ± 13.14 Nm) and the follicular phases (89.27 Nm ± 15.01 Nm). Both phases were not significantly different from the ovulatory phase (93.73 Nm ± 15.15 Nm). Although there was a trend toward an increase in concentric maximum extension strength across the cycle phases, with measurements of 121.50 Nm (median, range: 91 Nm ‐ 177 Nm) in the follicular phase, 125.00 Nm (median, range: 96 Nm ‐ 187 Nm) in the ovulation phase, and 127.5 Nm (median, range: 81 Nm ‐ 178 Nm) in the luteal phase, no statistically significant effect was found (Friedman test: *p* = 0.610; χ^2^(2) = 0.989; *W* = 0.022). These results are shown in Figure 3.

## 4 Discussion

The purpose of this study was to analyze the influence of the menstrual cycle on neuromuscular control and motor performance parameters associated with injury risk or athletic performance. Findings indicate that concentric maximum flexion strength and flexibility increased slightly across menstrual cycle phases, while jumping performance and balance control remained mostly constant.

### 4.1 Muscle Strength

Concentric maximum knee flexor and extensor strength increased slightly across the menstrual cycle, with significant effects observed only for the flexors. For the knee flexors ($\eta_{G}^{2}$ = 0.037) and the knee extensors (*W* = 0.022), the effect sizes were small, suggesting that the observed differences, although statistically detectable, are of limited functional relevance. However, the observed small increases in knee strength across sessions cannot be unequivocally attributed to menstrual cycle phase. Because participants were tested repeatedly, a session‐related learning or familiarization effect cannot be ruled out. Repeated exposure to maximal strength testing may have resulted in slight performance improvements independent of hormonal fluctuations. Thus, it remains unclear whether the detected main effects reflect true cycle‐related physiological changes or repeated testing adaptations. Overall, both strength outcomes tended to be slightly higher in the luteal phase and lower in the follicular phase. These findings are consistent with primary data from Thompson et al. (2021), who found no significant changes in maximal knee flexion or extension torque across menstrual cycle phases, despite rigorous phase verification using urinary LH tests and serum hormone measurements in a small sample of 10 females. Similarly, Weidauer et al. (2020) examined 22 healthy females with precise hormonal verification using both LH tests and blood analysis and observed only small differences in extension and flexion torque across the early‐follicular, ovulatory, and mid‐luteal phases, with statistically significant but minor effects (extension: 4% lower in early follicular vs. ovulation; flexion: 8% lower in early follicular vs. ovulation). For both strengths, the mid‐luteal phase was higher than the early‐follicular phase. Taken together, these findings suggest that menstrual cycle‐related fluctuations in maximal knee strength are likely small and of limited functional relevance, even when hormonal status is accurately determined. While hormonal such as estrogen‐related effects on muscle metabolism, mass, and strength (Chidi‐Ogbolu and Baar 2018; Dam et al. 2022; Iwańska et al. 2021), are often proposed as explanatory factors, our study did not assess serum hormones. Therefore, it remains unclear whether these increases are hormonally driven or attributable to other physiological or methodological factors, such as motivation, measurement variability, or daily fluctuations in performance.

The relationship between reduced muscle strength and injury risk through the menstrual cycle is under‐researched. While some authors discuss the influence of reduced muscle strength or imbalances for an increased risk of ACL (Hewett et al. 2007), hamstring (Opar et al. 2012), or ankle injuries (Delahunt and Remus 2019), most of the existing knowledge is based on a limited number of studies with athletes of both sexes. A closer analysis of studies that have differentiated between the sexes shows that strength deficits are likely to have a greater impact on the risk of injury in females than in males (Mason et al. 2022). Although injuries were not specifically analyzed, our findings seem to support a potential association between the observed increases in muscle strength and injury risk over the course of the menstrual cycle, possibly influenced by hormonal fluctuations. Knee muscle strength was the highest during the luteal phase which, reportedly, is also the phase with the lowest ACL injury risk (Smith et al. 2012). Thus, the lower maximum knee muscle torque in the early follicular phase could potentially act as a moderating factor for the risk of major knee injuries in females such as the ACL rupture. However, since some studies contradict these assumptions (Weidauer et al. 2020), more research is needed for better clarification.

### 4.2 Ankle Range of Motion

The menstrual cycle showed a statistically significant effect on ankle dorsiflexion range of motion (ROM), however, the effect size was small ($\eta_{G}^{2}$ = 0.010), and post‐hoc comparisons revealed no significant differences between phases. Mean values indicated a slight trend toward increased ROM from the follicular to the luteal phase. Previous studies have discussed the influence of estrogen on collagen structure, tendon elasticity, and ligament laxity (Chidi‐Ogbolu and Baar 2018; Eiling et al. 2007; Herzberg et al. 2017; Iwańska et al. 2021), hormonal concentrations were not measured in this study. Therefore, it cannot be determined whether the observed differences are related to hormonal fluctuations or reflect normal inter‐individual variability. Nevertheless, the small magnitude of changes observed in the present study aligns with the findings of Miyazaki and Maeda (2022), who examined knee extension ROM across menstrual cycle phases and reported only minor differences, with slightly increased flexibility in the luteal phase. They attributed the increase during the luteal phase primarily to changes in stretch tolerance rather than reductions in passive stiffness, suggesting that the apparent rise in flexibility may reflect habituation to pain rather than structural changes in muscle or connective tissue. Similarly, Sajjadi et al. (2025) observed slightly greater ankle plantarflexion ROM during the ovulation and luteal phase compared to the follicular phase in taekwondo athletes performing roundhouse kicks, although these differences were not statistically significant. However, given the small effect size, non‐significant post‐hoc results, and absence of direct hormonal data, our findings should be interpreted with caution. Taken together, these studies and the present results indicate that the menstrual cycle‐related fluctuations in joint ROM, whether at the knee or ankle, are subtle and unlikely to have functional implications for performance or injury prevention. Moreover, no clear evidence links increased ankle flexibility to sprain risk in females (Mason et al. 2022), likely due to the low number of studies with limited sample sizes or test specifications. Further research with hormonal and biomechanical monitoring is needed to clarify whether these small increases represent true physiological adaptations or normal inter‐individual variability.

### 4.3 Postural Control

Only minor non‐significant changes in the postural sway path were observed, suggesting that static balance control in healthy young females remains largely stable across menstrual cycle phases. The small effect size (*W* = 0.025) indicates minimal functional relevance. These findings are consistent with Reschechtko et al. (2023), who also reported no meaningful cycle‐related fluctuations in balance control, despite assessing participants at four distinct time points across the cycle with verified hormonal profiling via blood analyses. In their study, sessions were conducted in a fixed order starting on cycle day 2, which may have introduced potential learning effects but still revealed stable balance outcomes. By contrast, previous studies have shown partially divergent results. Yim et al. (2018) observed greater postural sway during the ovulation phase compared with the follicular phase, using an ovulation predictor kit to determine cycle phase. Conversely, Sawai et al. (2018), who employed a rigorous protocol combining serum hormone analyses, basal temperature, and urinary LH detection across five cycle phases, found a decrease in lateral sway from the follicular to the ovulatory phase, followed by an increase toward the late luteal phase. These discrepancies may stem from methodological variations in phase determination, task demands, and sample characteristics. A possible reason for these inconsistent findings lies in the specificity of balance assessments. Balance performance is highly task‐dependent and influenced by both the difficulty and familiarity of the task (Zech et al. 2014). Although all the aforementioned studies on balance control during the menstrual cycle measured standing postural sway, they varied in the standing conditions (two‐leg and single‐leg standing for different amounts of time and with or without visual feedback). Moreover, Kacem et al. (2021), who used a randomized phase order and verified cycle phases through both rectal temperature and urinary LH testing, found no significant menstrual cycle effect on static postural control. However, they did observe changes in dynamic balance performance, measured with the Y‐Balance Test (YBT). This suggests that more complex or demanding balance tasks might be required to detect potential hormonal or neuromuscular influences, particularly in young, physically active populations.

### 4.4 Jump Performance

No meaningful changes in jump performance (CMJ, SJ) across the menstrual cycle were observed in the present study, aligning with previous findings that suggest stable neuromuscular output across phases (Meignié et al. 2021). For the CMJ, the small effect size ($\eta_{G}^{2}$ = 0.007) indicates that differences between cycle phases were negligible in functional terms. In the same way, SJ performance also showed a small effect size (*W* = 0.015), further suggesting limited functional relevance. Overall, these findings support the notion that jump performance remains largely unaffected by menstrual cycle phase in healthy young females. Similarly, Julian et al. (2017), who verified menstrual cycle phases via serum estrogen and progesterone concentrations, found no significant differences in jump height between the follicular and luteal phases. These findings reinforce the notion that hormonal fluctuations alone may not meaningfully alter explosive lower‐limb performance. In contrast, García‐Pinillos et al. (2021) were able to show a significant effect of the menstrual cycle for the SJ with greater jump height in the follicular phase compared to the luteal phase. Their study included an initial familiarization session and non‐randomized testing across three phases based on cycle day counting from menstruation onset. While their results indicate phase‐related variation, the methodological approach, particularly the absence of hormonal verification, may have introduced potential misclassification bias. Furthermore, Dam et al. (2022) examined jump height across six hormonally verified menstrual phases (via blood sampling) and found only small, non‐significant effects in CMJ height. Importantly, these fluctuations were not associated with serum estrogen or progesterone levels, suggesting that the slight reductions observed around menstruation were more likely due to other circumstantial factors associated with the menstrual bleeding period that have limited the jump performance. Taking the current evidence as well as our findings into account, it can be assumed that the hormonal fluctuations during the menstrual cycle have no meaningful influence on the jumping performance of female athletes. To analyze possible associations with injury risk, it is probably more helpful to focus more on jump landing mechanisms than on the actual performance. It has been shown that females with ACL reconstruction significantly change their drop jump landing biomechanics throughout the menstrual cycle (Bell et al. 2014).

Although small differences were observed in some neuromuscular parameters, methodological limitations, such as fixed session order, lack of direct hormonal measurements, and approximate phase classification, mean that these findings should be interpreted with caution. Future studies should include multiple cycles, direct hormone measurements, and randomized session order to clarify the influence of menstrual cycle phases on neuromuscular performance.

## 5 Limitations

This study has several limitations that justify a careful interpretation of the results. First, only a single menstrual cycle was monitored to keep participant burden, drop‐outs, and costs manageable. Monitoring multiple cycles could provide more robust and reliable results by accounting for intra‐individual variability. Second, without the measurement of serum estrogen and progesterone levels, the determination of the different phases of the menstrual cycle can be less precise and prevent conclusions about hormonal influences on performance outcomes. According to Draper et al. (2018), the analysis of serum hormones and the use of urine LH tests together allow a precise classification of the menstrual cycle phases. Despite these limitations, ovulation indication using the LH urine test is a valid and established method. It is a non‐invasive and painless method and can help maintain the willingness to participate, especially with repeated measurements, thus reducing the drop‐out rate of eligible participants. Furthermore, studies by Schmalenberger et al. (2021) and Allen et al. (2016) support the use of urine LH tests as a cost‐effective and practical alternative to blood analysis, allowing for the accurate identification of menstrual cycle phases, although not the subphases. The timing of the measurement sessions in this study was based on self‐reported cycle tracking and LH surge detection, aiming to represent the early follicular, ovulatory, and mid‐luteal phases. This approach is comparable to previous studies examining menstrual cycle effects on neuromuscular performance (e.g., Abt et al. 2007; Blake et al. 2016; Erden et al. 2022; Ham et al. 2020; Harriell et al. 2010; Khowailed et al. 2022; Miyazaki and Maeda 2022). However, as blood hormone concentrations were not assessed, it is likely that the actual estradiol peak was missed (Elliott‐Sale et al. 2021). This limitation should be considered when interpreting between‐phase comparisons. According to Elliott‐Sale et al. (2021), our classification (follicular, ovulation, luteal) approximately corresponds to phases 1, 3, and 4, but without direct hormonal verification. This may partly explain discrepancies between our findings and those of previous studies that applied different phase definitions or measured hormone concentrations directly. Using a standardized framework may help improve comparability across studies and clarify whether subtle changes are phase‐dependent or reflect individual variability. Finally, because session order was determined by cycle timing (fixed sequential order: follicular, ovulatory, luteal phases), full randomization across sessions was not feasible. Even when measurements were rescheduled to the next cycle due to an absent LH surge, the sequential order remained unchanged. As session order and menstrual cycle phase are fully confounded, separate statistical testing for order effects is not possible and would not provide additional information beyond the phase comparisons already conducted. Therefore, potential learning or familiarization effects across testing sessions cannot be ruled out and may have influenced the results. Additionally, the jump tests focused only on jump height and excluded landing mechanics, which could provide further insights into injury risk.

## 6 Conclusion

This study examined the effects of menstrual cycle phases on neuromuscular control and motor performance in healthy young female participants. Small increases were observed in maximum knee flexor muscle strength and ankle dorsiflexion range of motion (ROM) across the menstrual cycle, whereas jumping performance and postural control remained stable. Given the small effect sizes, and the limited number of significant post‐hoc comparisons (only concentric knee flexor strength), as well as the absence of direct hormonal measurements, these findings should be interpreted with caution. Overall, the results suggest that neuromuscular and motor performance parameters are largely stable throughout the menstrual cycle, with only subtle fluctuations that are unlikely to have meaningful functional implications for performance or injury risk. Nonetheless, individual differences may exist, and monitoring athletes across their cycles could still be valuable for personalized training and load management. These findings align with the broader literature suggesting small and inconsistent phase‐related effects on performance.

## 7 Practical Implications

— Coaches and athletes should be aware that small, individual differences in strength and flexibility may occur across menstrual cycle phases.
— Since most performance parameters appear to remain stable, training programs do not require major adjustments but may benefit from flexible scheduling during menstruation or perceived low‐performance phases.
— Promoting awareness and open communication about the menstrual cycle can improve athlete comfort, motivation, and training adherence, without implying necessary changes to training content.

## Author Contributions

The contributions and responsibilities of each author can be summarized as follows: M.S. and N.O. collected the data and prepared the data for analysis, M.S. performed the statistical analysis, M.S. wrote the original draft, A.Z. and N.O. reviewed and edited the manuscript. All authors have read and agreed to the published version of the manuscript.

## Funding

The authors have nothing to report.

## Ethics Statement

This study was approved by the Ethical Commission of the Faculty of Social and Behavioral Science of the Friedrich‐Schiller University of Jena, approval number FSV 22/010. During the study process, the authors followed the rules of the Helsinki Declaration.

## Conflicts of Interest

The authors declare no conflicts of interest.

## Supporting information


**Table S1:** Results of the parameters recorded over the three phases of the menstrual cycle of the 22 participants.

## Data Availability

The data that support the findings of this study are available on request from the corresponding author. The data are not publicly available because of privacy or ethical restrictions.
