# Supplementary material for: Neuromuscular Control and Motor Performance Across the Menstrual Cycle in Physically Active Young Females
Source: Eur J Sport Sci. 2026 Apr 11;26(5):e70174. doi: 10.1002/ejsc.70174 (PMC13070097; doi:10.1002/ejsc.70174)
Supplement: Supplementary file 1 — Table S1: Results of the parameters recorded over the three phases of the menstrual cycle of the 22 participants. [file EJSC-26-e70174-s001.docx]

**Table list (Appendix)**

Table 1. *Results of the parameters recorded over the three phases of the menstrual cycle of the 22 participants*

|  |  |  |  |  |  |  |
| --- | --- | --- | --- | --- | --- | --- |
|  |  | **Follicular phase** | **Ovulation phase** | **Luteal phase** | **ANOVA** | **Friedman test** |
|  | Body weight [kg] | 65.01 ± 6.37 | 64.62 ± 6.36 | 64.58 ± 6.48 | df=2,42 F=2.36 p=0.107 $\eta_{G}^{2}$ <0.001 |  |
|  | Body Mass Index [kg/m²] | 22.97 ± 1.89 | 22.83 ± 1.87 | 22.82 ± 1.94 | df=2,42 F=2,37 p=0.106 $\eta_{G}^{2}$=0.001 |  |
|  | Total body water [l] | 36.91 ± 3.71 | 37.11 ± 4.35 | 36.77 ± 3.99 | df=2,42 F=0.61 p=0.511 $\eta_{G}^{2}$=0.001 |  |
|  | Countermovement Jump [cm] | 24.77 ± 3.47 | 25.36 ± 3.48 | 24.74 ± 3.80 | df=2,42  F=1.55 p=0.224  $\eta_{G}^{2}$=0.007 |  |
|  | Squat Jump [cm] | 22.47 ± 2.88 | 22.86 ± 3.13 | 23.01 ± 3.44 |  | df=2  χ²=0.659 p=0.719 W=0.015 |
|  | Static Body Sway [mm] | 509.32 ± 174.60 | 471.21 ± 117.52 | 494.51 ± 134.18 |  | df=2  χ²=1.091 p=0.580 W=0.025 |
|  | Ankle dorsiflexion Range of motion [°] | 36.68 ± 5.78 | 37.50 ± 6.33 | 38.09 ± 6.05 | df=2,42  F=3.40  p=0.043 *^b^ $\eta_{G}^{2}$=0.010 |  |
|  | Knee extension strength [Nm] | 125.55 ± 25.16 | 128.64 ± 26.92 | 130.00 ± 26.98 |  | df=2  χ²=0.989 p=0.610 W=0.022 |
|  | Knee flexion strength [Nm] | 89.27 ± 15.01 | 93.73 ± 15.15 | 95.91 ± 13.14 | df=2,42 F=6.61  p=0.003 *^a^ $\eta_{G}^{2}$=0.037 |  |
|  |  |  |  |  |  |  |

**Note.** Mean ± standard deviation, df indicates the degrees of freedom, F indicates the F-value from the Anova (analysis of variance), p indicates the probability of accepting the null hypothesis (H0), $\eta_{G}^{2}$ indicates the effect size using the generalized eta squared, χ² indicates the chi-square statistic from Friedman test, W indicates the effect size using Kendall’s W; * indicates the significant effect in the ANOVA, ^b^ the post-hoc test shows no significance between the phases, ^a^ the post-hoc test shows a significant effect between the follicular and luteal phase (p=0.004).
